# Airway Microbial Diversity is Inversely Associated with Mite-Sensitized Rhinitis and Asthma in Early Childhood

**DOI:** 10.1038/s41598-017-02067-7

**Published:** 2017-05-12

**Authors:** Chih-Yung Chiu, Yi-Ling Chan, Yu-Shuen Tsai, Ssu-An Chen, Chia-Jung Wang, Kuan-Fu Chen, I.-Fang Chung

**Affiliations:** 1 Department of Pediatrics, Chang Gung Memorial Hospital at Keelung, and Chang Gung University, Taoyuan, Taiwan; 2 Division of Pediatric Pulmonology, Department of Pediatrics, Chang Gung Memorial Hospital at Linkou, and Chang Gung University, Taoyuan, Taiwan; 3 Department of Emergency Medicine, Chang Gung Memorial Hospital at Linkou, Taoyuan, Taiwan; 4 The Institute of Biomedical Informatics, National Yang-Ming University, Taipei, Taiwan; 5 The Center for Systems and Synthetic Biology, National Yang-Ming University, Taipei, Taiwan

## Abstract

Microbiota plays an important role in regulating immune responses associated with atopic diseases. We sought to evaluate relationships among airway microbiota, serum IgE levels, allergic sensitization and their relevance to rhinitis and asthma. Microbial characterization was performed using Illumina-based 16S rRNA gene sequencing of 87 throat swabs collected from children with asthma (n = 32) and rhinitis (n = 23), and from healthy controls (n = 32). Data analysis was performed using QIIME (Quantitative Insights Into Microbial Ecology) v1.8. Significantly higher abundance of Proteobacteria was found in children with rhinitis than in the healthy controls (20.1% vs. 16.1%, *P* = 0.009). Bacterial species richness (Chao1 index) and diversity (Shannon index) were significantly reduced in children with mite sensitization but not in those with food or IgE sensitization. Compared with healthy children without mite sensitization, the mite-sensitized children with rhinitis and asthma showed significantly lower Chao1 and Shannon indices. *Moraxella* and *Leptotrichia* species were significantly found in the interaction of mite sensitization with rhinitis and asthma respectively. Airway microbial diversity appears to be inversely associated with sensitization to house dust mites. A modulation between airway dysbiosis and responses to allergens may potentially cause susceptibility to rhinitis and asthma in early childhood.

## Introduction

The occurrence of asthma and allergic diseases has increased in the past few decades and remains a major health issue for children^1^. Asthma is a complex disease caused by the combined effects of many genetic factors interacting with environmental factors^2^. Accumulating evidence suggests that exposure to environmental microbiota has a protective effect against childhood asthma^3, 4^. Candidate gene-based studies in asthma patients have also identified genes such as *IL-13* and *FcεRI-β*, which are known to modify microbial pattern recognition receptors of the innate immune system^5^. These studies collectively indicate the importance of bacterial microbiota in gene-environment interactions in patients with asthma and other potential allergic diseases.

Interaction between the mucosal microbiota and immune cells is important in the development of the immune system^6^. The microbiota mediates mechanisms of immunological tolerance and is essential for regulating mucosal inflammation. Early exposure to microorganisms may program infant immunity to a type 2T helper (Th2) bias that is related with atopy and asthma development^7, 8^. Environmental allergens also trigger Th2 cell-mediated immune responses with recruitment of immunoglobulin E (IgE) antibody-producing B cells, mast cells and eosinophils^6^. Early sensitization to food has been identified as a risk factor for developing allergic airway diseases, whereas sensitization to inhalant allergens appears to be more specific to the development of rhinitis and asthma in early childhood^9, 10^. Although asthma and rhinitis are frequently associated with atopy with preferential sensitization to aeroallergens, their clinical manifestations present at quite different airway levels. Several studies in humans and animal models of diseases have emphasized that an altered microbiota is related to a higher prevalence of atopic diseases and asthma^11, 12^. However, few studies have focused on the association between microbiota composition, especially in the airways, and allergen sensitization and allergic airway diseases such as atopic rhinitis and asthma in early childhood.

A detailed understanding of the complexity of airway microbial profiles during early childhood will likely provide much needed clinical insights into this aspect of airway diseases. The oropharynx is constantly exposed to microbes from both the upper and lower respiratory tract, and these microbes may be related to atopic airway diseases including allergic rhinitis and bronchial asthma. The aim of this study was to examine the oropharyngeal microbial profiles in patients with childhood rhinitis, asthma and healthy controls. The relationship between bacterial composition and community diversity and atopic indices including total IgE levels and allergen sensitization were assessed; their relevance to rhinitis and asthma was also examined.

## Results

### Population Characteristics

Eighty-seven subjects were enrolled into this study, including 32 children with asthma, 23 children with allergic rhinitis, and 32 healthy controls. The baseline characteristics of children with asthma, those with rhinitis, and the healthy controls are shown in Table 1. The average age of the subjects was 4.4 years (range: 3.7–4.9 years) and the mean age was higher in the healthy children than in the children with asthma or rhinitis (4.2 ± 0.5 vs. 4.4 ± 0.4 vs. 4.6 ± 0.3, respectively; *P* = 0.001). Atopic indices including total IgE levels and allergen sensitization to mites and food were significantly higher in children with asthma and rhinitis than in the healthy controls. There was no difference other characteristics such as sex, maternal atopy, passive smoking, older siblings and household income between the three groups.

**Table 1:** Epidemiologic characteristics of the 87 children investigated in this study.

Characteristics	Asthma	Rhinitis	Control	*P*-value
	(n = 32)	(n = 23)	(n = 32)	
Age (yr)	4.2 ± 0.5	4.4 ± 0.4	4.6 ± 0.3	0.001
Sex, male	13 (40.6%)	10 (43.5%)	14 (43.8%)	0.963
Maternal atopy	19 (59.4%)	10 (43.5%)	13 (40.6%)	0.281
Passive smoking	11 (42.3%)	7 (31.8%)	10 (31.2%)	0.636
Older siblings	7 (46.7%)	13 (59.1%)	15 (46.9%)	0.636
Household income				0.119
Low, ≤ 500,000 NTD	8 (33.3%)	10 (41.7%)	6 (25.0%)	
Medium, 500,000-1,000,000 NTD	9 (40.9%)	6 (27.3%)	7 (31.8%)	
High, >1,000,000 NTD	13 (40.6%)	17 (53.1%)	2 (6.2%)	
Total IgE, kU/L	281.4 ± 365.6	255.2 ± 312.9	53.5 ± 61.3	<0.001
IgE > 100 kU/L	18 (62.1%)	13 (56.5%)	6 (18.8%)	0.001
Mite sensitization	19 (65.5%)	15 (68.2%)	11 (34.4%)	0.016
Food sensitization	15 (57.7%)	13 (59.1%)	9 (28.1%)	0.029

Data shown are mean ± SD or number (%) of patients as appropriate. yr, year; NTD, New Taiwan Dollar; IgE, immunoglobulin E.

### Bacterial Composition and Abundance Categorized by Allergic Airway Diseases

**Figure 1:**
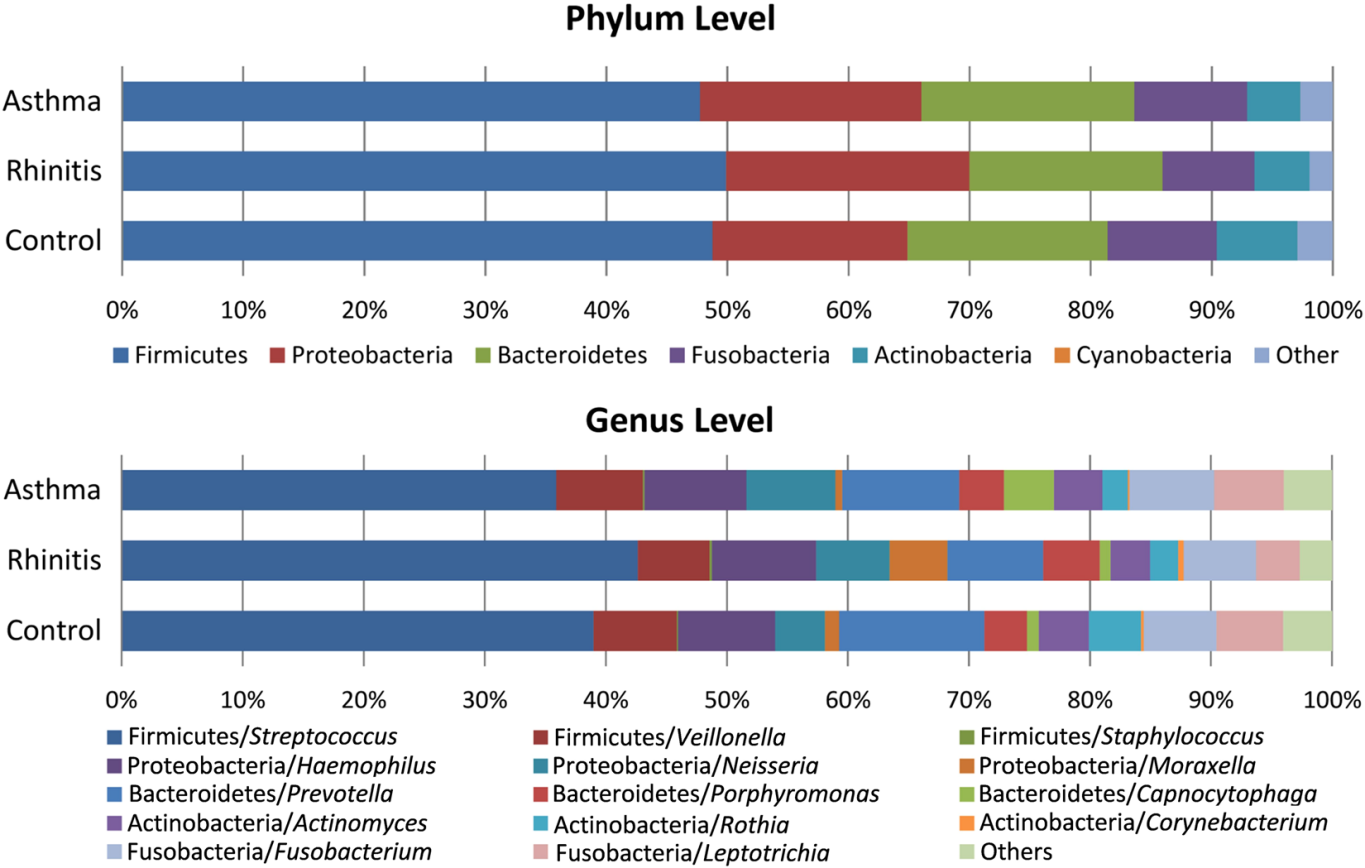
Bacterial composition and abundance at phylum and genus levels. Each row represents the distribution of the five most abundant phyla and their three most common genera in children with asthma, rhinitis and healthy controls. The color of each cell indicates the relative abundance of bacterial phyla and genera.

A range of reads from 38,348 to 394,673 was obtained per sample, and a total of 2,811 OTUs were obtained from all the subjects. Rarefaction curves showed that a plateau of species richness (up to 800 OTUs) was achieved in around 38,000 reads per samples (Supplementary Fig. S1). In order to control sample heterogeneity, randomly 38,000 reads was used as the minimum sampling depth to capture diversity. The bacterial composition and abundance at the phylum and genus levels are shown in Fig. 1. Taxonomic classification showed a high prevalence of members of the phylum Firmicutes (48.8% of the total number of sequences obtained) followed by those of the phyla Proteobacteria (18.2%), Bacteroidetes (16.7%), Fusobacteria (8.6%), Actinobacteria (5.2%), and others. *Streptococcus* (26.0%), *Haemophilus* (5.5%) and *Prevotella* (7.6%) were the most common genera in the phylum Firmicutes, Proteobacteria, and Bacteroidetes respectively.

### Differential Abundance Analysis of the Microorganisms for Rhinitis and Asthma

Differences in the abundance of members of different phyla and genera among the children with asthma, those with rhinitis, and the healthy controls are shown in Table 2. At the phylum level, a significantly higher prevalence of Proteobacteria was found in children with rhinitis than in the healthy controls (20.1% vs. 16.1%, *P* = 0.009). Furthermore, the abundance of members of the genera *Haemophilus*, *Neisseria*, and *Moraxella* of the phylum Proteobacteria in children with rhinitis was significantly increased.

**Table 2:** Differences and comparisons of bacteria in phyla and genera among children with asthma, rhinitis and healthy controls.

Phylum/Genus	Asthma (n = 32)	Rhinitis (n = 23)	Control (n = 32)	Rhinitis vs. Control	Asthma vs. Control
	mean ± SD (%)	mean ± SD (%)	mean ± SD (%)	FDR *P*-value	FDR *P*-value
Bacteroidetes/*Porphyromonas*	2.35 ± 2.20	3.85 ± 3.77	2.96 ± 2.83	0.015	
Firmicutes/*Moryella*	0.66 ± 1.59	0.17 ± 0.21	0.57 ± 1.00	0.020	
Fusobacteria/*Fusobacterium*	4.83 ± 4.54	5.27 ± 4.37	5.07 ± 5.22	0.048	
Proteobacteria/*Aggregatibacter*	0.62 ± 1.67	0.28 ± 0.91	0.62 ± 1.25	0.020	
Proteobacteria/*Haemophilus*	5.26 ± 4.57	5.98 ± 3.54	5.40 ± 5.54	0.040	
Proteobacteria/*Neisseria*	4.33 ± 4.84	5.08 ± 4.60	2.86 ± 2.42	0.009	
Proteobacteria/*Moraxella*	0.62 ± 2.41	1.95 ± 4.14	0.50 ± 1.17	0.030	
Firmicutes/*Butyrivibrio*	0.03 ± 0.07	0.03 ± 0.05	0.09 ± 0.19	0.019	0.030
Firmicutes/*Parvimonas*	0.19 ± 0.31	0.80 ± 1.25	0.48 ± 0.75		0.020
Firmicutes/*Selenomonas*	0.61 ± 0.84	0.36 ± 0.50	0.57 ± 0.72		0.020

Data shown are mean ± SD of relative abundance of bacteria. The percent of total numbers of sequences are shown for each split level. Only taxonomic classification with more than 100 sequences or 0.01% of total sequences, and statistically significant differences are shown. FDR-adjusted *P*-values are calculated by metagenomeSeq differential abundance tests in the R software using subsampled microbial communities.

### Bacterial Richness and Diversity Categorized by Atopic Indices

**Figure 2:**
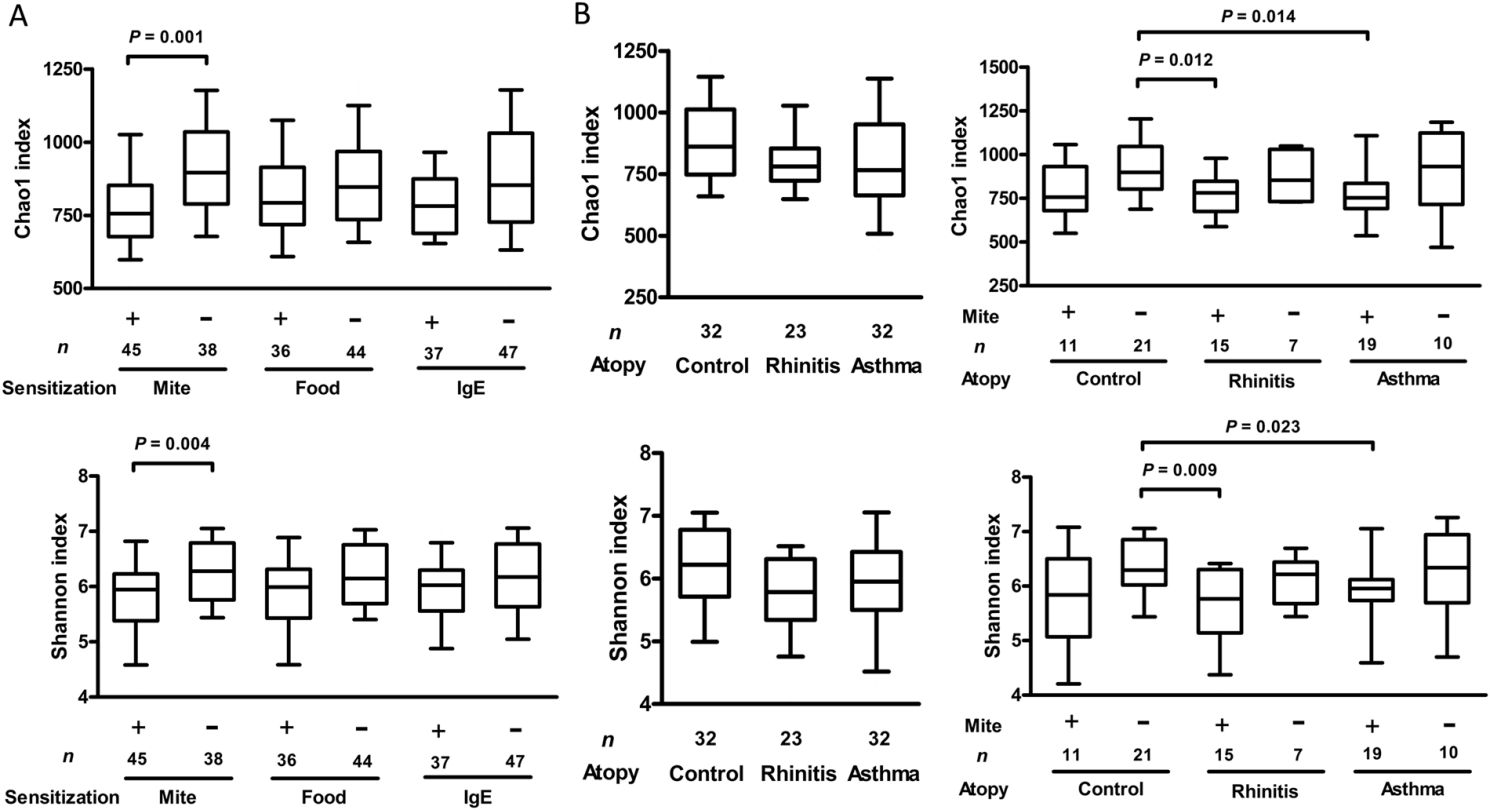
Estimates of bacterial richness and diversity. (**A**) Differences and comparisons of species richness (Chao1 index) and diversity (Shannon index) for atopic indices including allergen sensitization to mite and food, and IgE sensitization (≥100 kU/L). (**B**) Differences and comparisons of species richness and diversity among topic diseases, and combined with and without mite sensitization. Species richness calculated as the Chao1 index. Species diversity calculated as the Shannon index. The box-plot shows the median and the 10^th^, 25^th^, 75^th^ and 90^th^ percentile. Only significant *P* values obtained by the Mann-Whitney test are shown.

The Chao1 index, a measure of species richness, and the Shannon index, a measure of species diversity, were calculated and analyzed. Bacterial richness and diversity were not significantly different with regard to the age, sex, maternal atopy, passive smoking, older siblings, and household income characteristics of the patients. Figure 2A shows the species richness and diversity categorized by atopic indices. The Chao1 and Shannon indices were significantly reduced in children with mite sensitization but not in those with food or IgE sensitization. Differences in the bacterial richness and diversity among children with asthma, those with rhinitis and the healthy controls are shown in Fig. 2B. Relatively lower Chao1 and Shannon indices were found in children with asthma and rhinitis than in the healthy controls, but these differences were not significant. However, the Chao1 and Shannon indices in the mite-sensitized children with rhinitis and asthma were significantly lower than those in the healthy children without mite sensitization.

Beta diversity statistics using the Principal Coordinate Analysis (PCoA) and non-metric multidimensional scaling (NMDS) revealed no significant differences in the microbial communities cluster patterns with regard to food, IgE sensitization and allergic airway diseases. However, a good clustering was observed when comparing between with/without mite sensitization (Supplementary Fig. S2).

### Microorganisms Associated with Rhinitis and Asthma over Mite Sensitization

**Figure 3:**
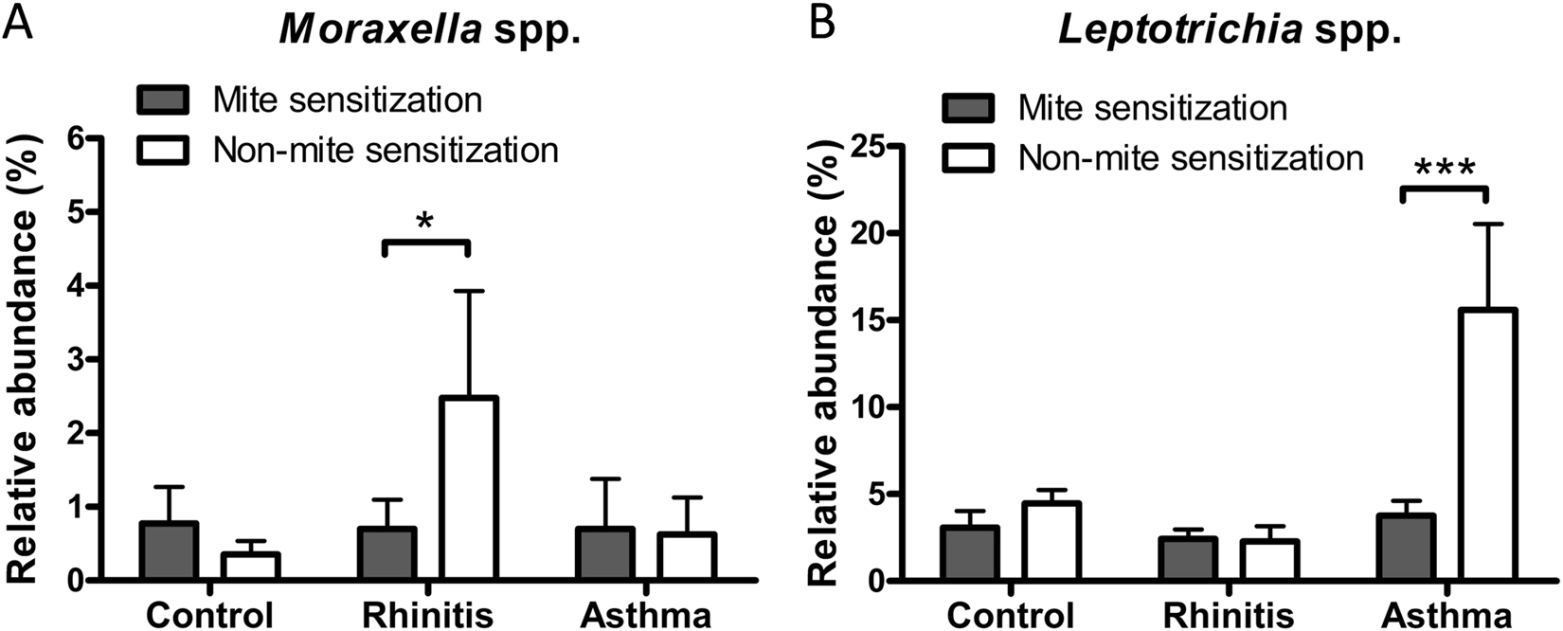
Genera *Moraxella* and *Leptotrichia* involving in the interaction between mite sensitization and allergic airway diseases. *Moraxella* spp. (**A**) and *Leptotrichia* spp. (**B**) showing significantly increased abundance in rhinitis and asthma without mite sensitization, respectively.

Airway microorganisms associated with mite sensitization for rhinitis and asthma was studied in a linear mixed model for repeated measures. Microorganisms identified to be involved in the interaction between allergic airway diseases and mite sensitization are shown in Table 3. Members of the genera *Moraxella* and *Leptotrichia* were significantly found in rhinitis and asthma interacting with mite sensitization, respectively. *Prevotella* spp. was significantly associated with sensitization to house dust mites but not rhinitis or asthma. Furthermore, both *Moraxella* spp. and *Leptotrichia* spp. were more abundant in children without mite sensitization (Fig. 3).

**Table 3:** Microbial profiles identified in the interaction of mite sensitization with rhinitis and asthma.

Genus	Abundance (%)	Asthma vs. Rhinitis vs. Control			Rhinitis vs. Control			Asthma vs. Control		
		*P* Interaction	*P* Disease	*P* Mite	*P* Interaction	*P* Disease	*P* Mite	*P* Interaction	*P* Disease	*P* Mite
*Leptotrichia*	3.90 ± 3.99	**<0.001**	**<0.001**	**<0.001**	0.993	0.150	0.260	**<0.001**	**<0.001**	**<0.001**
*Moraxella*	0.93 ± 2.71	**0.046**	**0.043**	0.238	**0.029**	0.077	0.210	0.494	0.330	0.544
*Neisseria*	3.99 ± 4.10	0.284	**0.003**	0.122	0.125	**0.001**	0.120	0.644	0.155	0.410
*Selenomonas*	0.53 ± 0.72	0.190	**0.042**	**0.010**	0.650	0.448	0.174	0.132	0.080	**0.029**
*Mogibacterium*	0.12 ± 0.14	0.185	0.850	**0.035**	0.253	0.931	0.258	0.448	0.511	**0.011**
*Butyrivibrio*	0.51 ± 0.12	0.526	0.447	**0.043**	0.597	0.278	0.122	0.815	0.863	**0.044**
*Prevotella*	7.77 ± 5.87	0.543	0.051	**0.030**	0.828	0.115	0.082	0.496	0.214	**0.047**

Data shown are mean ± SD of relative abundance of bacteria. *P-*value was adjusted for maternal atopy, passive smoking, older siblings, household income, child’s sex and age. All *P*-values < 0.05, which is in bold, are significant.

## Discussion

The association of airway microbiota with allergen sensitization and allergic airway diseases has not been well determined so far. This study demonstrated that the existence of an inverse association between airway microbial diversity and sensitization to house dust mites. Dysbiosis of particular subsets of the airway microbiota may take part in allergic reactions in response to allergen exposure, which can potentially cause susceptibility to rhinitis and asthma in early childhood.

Microbes associated with humans play an essential role in immunity and health, and microbial community composition has been linked with several different diseases^13, 14^. The first study to examine airway microbiota using culture-independent techniques revealed that airways are not sterile and possess their own specific microbiota^15^. Although the dispersal of oral commensals has been shown to be the dominant source of organisms shaping airway microbiota, the composition of the lower airway microbiome does not derive entirely from the oral cavity^15, 16^. Despite this, the oropharynx is constantly exposed to microbes from both the upper and lower respiratory tract, where may represent the microbes related to airway diseases including allergic rhinitis and asthma^17, 18^.

Several studies have reported associations between gross metrics of bacterial community composition and asthma^19^. In allergy and asthma, a reduced airway microbial diversity has been hypothesized to increase the risk of allergic manifestations^20^. As in this study, a relatively lower bacterial richness and diversity in the airways was found in children with childhood rhinitis and asthma than in the healthy controls. However, the bacterial diversity colonizing the airways has been found to be greater in adult asthmatic compared with healthy subjects^21^. Although this inconsistency may be a result of differences in sampling site, age, and the spectrum of disease severity in the study populations, this discrepancy highlights potential differences in airway microbiota affecting asthma between adults and children.

The upper respiratory tract is considered the point of entry for aeroallergens and commensal bacteria into the airways through microaspiration^18^. Exposure and sensitization to allergens are the most important risk factors for the development of allergic airway diseases^22^. Bacterial exposure has been reported to modulate immunity to against atopic asthma by suppressing Th2 immune responses, which are related to allergic reactions in response to allergens^23^. In this study, low airway microbial diversity appeared to be associated with increased mite sensitization. In addition, it was also found to be lower in mite-sensitized children with rhinitis and asthma than in healthy children without mite sensitization. These findings suggest alterations in airway microbiota induce or suppress systemic immune tolerance to inhalant antigens, which may be attributed to the link between the microbiota composition and atopic disease.

The diversity of microbial species, rather than exposure to one particular microbial component, is particularly protective^24^. However, exposure to different types of bacteria may have different effects in allergic diseases. In this study, members of the phylum Proteobacteria, including the genera *Haemophilus*, *Neisseria*, and *Moraxella*, were found numerically to be more abundant in the airways of children with rhinitis than in the healthy controls. Clinically, these bacteria are the most common pathogens in acute and chronic sinusitis and otitis media, which are usually complicated by poorly controlled symptoms of allergic rhinitis^25^. In particular, the genus *Moraxella* has been shown to be involved in the interaction between mite sensitization and rhinitis, suggesting that *Moraxella* spp. may not only contribute to poor rhinitis control, but also play a role in modulating allergen sensitization that is involved in allergic rhinitis.

Despite a significant relationship between microbiota and asthma, no particular subsets of the microbiota have been consistently found to be associated with asthma. *Neisseria* species have been identified to be abundant in asthmatics and related to the degree of eosinophilic inflammation^26^. In this study, *Neisseria* spp. were found to be more abundant in children with rhinitis but not asthma without association with allergen sensitization. The presence of members of the phylum Bacteroidetes, especially *Prevotella* spp., in the airways has been reported to be protective against asthma in older children and adults^15^. However, in this study, *Prevotella* spp. was found to be significantly associated with sensitization to house dust mites but not rhinitis or asthma. Similarly to *Prevotella* spp., *Butyrivibrio* spp. and *Selenomonas* spp. appeared to be associated with asthma because of their strong association with mite sensitization. These results indicate that a modulation between microbial dysbiosis and responses to allergens in the airways may potentially cause differences in susceptibility to childhood rhinitis and asthma.

*Leptotrichia* species are typical anaerobic, gram-negative bacilli, which are primarily part of the normal flora in the mouth. In this study, *Leptotrichia* spp. were not only strongly associated with mite sensitization but were also linked with asthma. In particular, *Leptotrichia* spp. appeared to be more abundant in children without mite sensitization, supporting the results from one other birth cohort study that reported an inverse association between the abundance of gram-negative bacteria and allergic sensitization^27^. Although the clinical importance of *Leptotrichia* spp. remains unclear, the involvement of *Leptotrichia* spp. in immune responses to allergens may particularly explain the pathogenesis of asthma in early childhood.

One major limitation of this study is a relatively small sample size resulting limited statistical power for the subanalyses. Furthermore, this study’s relatively short follow-up period may make the diagnosis of subclinical atopy be missed. The presence of diverse microorganisms across different sites and the dissimilarities in microbial compositions in a wide range of age groups are two common limitations for microbiome analysis. The strength of the present study, however, lies in its accurate sample collection performed by the same physician for a consistent sampling site. A comparison of the microbiota in children with a very small age difference also makes our results valid and potentially important.

In conclusion, the analysis of airway microbiota provides new insights into its pathogenic potential and implication on health and in allergic airway diseases. The microbial diversity in airways appears to be inversely associated not only with allergen sensitization, but also with rhinitis and asthma in mite-sensitized children, indicating that the interactions between airway microbiota and allergic reactions in response to allergen exposure may play an important role in allergic airway diseases. *Moraxella* spp. and *Leptotrichia* spp. were found to significantly contribute to this interaction, suggesting that a modulation between particular subsets of airway dysbiosis and allergen responses could potentially cause susceptibility to rhinitis and asthma. Further studies with functional analysis, however, are required to investigate these associations more comprehensively.

## Methods

### Study Design

A case-control study was designed to investigate the airway microbiota profiles in patients with asthma or rhinitis in early childhood and in healthy controls. Children (ages: 3–5 years) who diagnosed with asthma alone or rhinitis alone for the first time, and healthy controls from August 1, 2013 to July 31, 2015 were recruited for this study. The physician-diagnosed phenotypes of atopic diseases were evaluated by the same pediatric pulmonologist at the outpatient clinics. Asthma was diagnosed as ever having asthma, with the occurrence of recurrent wheeze in the last 12 months, or current use of asthma medication, based on the guidelines of the Global Initiative for Asthma^28^. Allergic rhinitis was diagnosed as having symptoms such as sneezing, nasal congestion, itching, and rhinorrhea in the last 12 months^29^. Healthy controls without a history of asthma or other atopic conditions or infections were enrolled and paired. Children who present with a combination of asthma and rhinitis when diagnosed were excluded. Children suffered an upper airway infection recently or chronic viral infection mimicking atopic diseases were also excluded. This study was approved by the Ethics Committee of Chang Gung Memory Hospital (No. 102-1315B). All experiments in this study were performed in accordance with the relevant guidelines and regulations and written informed consent was obtained from the parents or guardians of all the study subjects.

### Data Collection

Detailed information on potential confounding variables for atopic diseases including child’s age, sex, maternal atopy, passive smoking, older siblings at birth, and household income (low, ≤500,000 NTD; medium, 500,000–1,000,000 NTD, and high, >1,000,000 NTD) were recorded and analyzed. The measurement of total and allergen-specific serum IgE levels was also performed and the results were recorded. Allergen-specific IgE levels were determined using a commercial assay for IgE (ImmunoCAP Phadiatop Infant; Phadia) for a mix of three most common food allergens (egg white, milk, and wheat) and two most common inhalant causing sensitization in more than 95% of children in Taiwan (*Dermatophagoides pteronyssinus* and *Dermatophagoides farina*)^30, 31^. Values of a total IgE level exceeding 100 kU/L was considered indicative of IgE sensitization^32^. Allergic sensitization was defined as ImmunoCAP Phadiatop Infant values ≥0.35  kU/L^33^.

### Sample Collection and Storage

Throat swabs were collected by a physician using sterile cotton swabs before inhaled or nasal administration of corticosteroids for regular daily treatment. None of the subjects had received antibiotics for at least four weeks prior to the sampling. Sampling was performed carefully without touching any surface (i.e. tongue, mouth, and teeth) other than the oropharynx and by using a tongue depressor. Each swab was rubbed at least three times around the oropharynx with swab rotation without interruption. After sampling, the swab was immediately placed back into the collection tube and stored at −80 °C until further use.

### DNA Extraction, 16S rRNA Gene Amplification, and Sequencing

Bacterial DNA was extracted from the throat swab using a FastDNA Spin Kit for Soil (MP Biomedical, Solon, OH, USA) following the manufacturer’s instructions. DNA was extracted with 70 µL of DNase/Pyrogen-Free Water and the purity was quantified by measuring the absorbance at 260 and 280 nm with a spectrophotometer (Nanodrop 1000; Thermo Scientific, Waltham, MA, USA). All samples had an A260-to-A280 absorbance ratio between 1.8 and 2.1. Polymerase chain reaction (PCR) was used to amplify the variable region 3 (V3) to V4 of the gene that encodes for 16 S rRNA in bacteria. The V3-V4 region of the 16S rRNA gene was amplified by complementing both Bakt_341F (CCTACGGGNGGCWGCAG) and Bakt_805R (GACTACHVGGGTATCTAATCC) with sample-specific barcodes^34, 35^. The amplicons were quantified and standardized to the same concentration using a Qubit 3.0 fluorometer (Thermo Fisher Scientific). PCR products with a concentration of less than 1.0 ng/µL were excluded from the study^36, 37^. Amplicon pyrosequencing was performed on the Illumina MiSeq platform (Illumina, Inc., San Diego, CA, USA) using the MiSeq reagent kit v3 at 600 cycles, according to the manufacturer’s instructions. The sequence data and mapping file for all the samples included in this study have been deposited in Figshare (http://dx.doi.org/10.6084/m9.figshare.3839838).

### Sequence Processing and Data Analysis

Illumina sequencing was performed by using paired-end 300 bp reads and the entire target was assembled with a central overlap of 50 bp by using PANDASeq^38^. Reads less than 100 nucleotides or lacking a correct primer and chimeric sequences were removed^39^. Data analysis was performed using the software “Quantitative Insights into Microbial Ecology” (QIIME)^40^. Assembled sequences were clustered into Operational taxonomic Units (OTUs) using UCLUST at 97% sequence identity^41^, and taxonomy classification was assigned based on full-length 16 S rRNA gene database, Greengenes^42^. Any sequences present once (singletons) or in only one sample were filtered out.

Bacteria diversity was analyzed with QIIME v1.8 software and rarefaction curves based on the number of species were generated for each sample from randomized OTU draws. Richness of each sample was calculated with the Chao1 index^43^ and diversity accounting for both relative abundance and evenness was evaluated with Shannon index^44^. Beta diversity was calculated between groups at OTU genus level using QIIME, and Phyloseq package and Adonis in R. Principal Coordinate Analysis (PCoA) plot in conjunction with unweighted UniFrac and non-metric multidimensional scaling (NMDS) plot in conjunction with RLB distance measure were produced to show clustering between groups^45, 46^. Rare OTUs were defined as less than 0.01% of the reads in a given sample, and were removed if more than 50% of all samples^47, 48^. Abundance differences between allergic airway diseases and healthy controls were tested using metagenomeSeq Bioconductor package for the R statistical computing environment^49^. A novel normalization method avoids bias due to uneven sequencing depth and a zero-inflated Gaussian distribution mixture model removes testing bias resulting from undersampling of the microbial community. A false discovery rate of 5% was applied to correct for multiple tests^50^.

### Statistical Analysis

Differences and comparisons of the baseline characteristics among the asthma patients, rhinitis patients, and the healthy controls were performed using univariable parametric and non-parametric tests such as analysis of variance (ANOVA) and Kruskal-Wallis test respectively. All continuous variables, such as species richness and diversity were analyzed using the Mann-Whitney test for comparison between two groups and the Kruskal-Wallis test for comparisons between three groups. Repeated measures two-way analysis of variance (ANOVA) with first order autoregressive covariance structure was used to study the interaction between allergen sensitization and the relative abundance of the microorganisms for allergic airway diseases, and adjusted for confounding variables including age, sex, maternal atopy, passive smoking, older siblings, and household income. Statistical analysis was performed by using the Statistical Package for the Social Sciences (SPSS Statistics for Windows Version 20.0; Armonk, NY, USA). All statistical hypothesis tests were two-tailed and a *P*-value < 0.05 was considered significant.

## Electronic supplementary material

Supplementary Information
