# Supplementary material for: Airway Microbial Diversity is Inversely Associated with Mite-Sensitized Rhinitis and Asthma in Early Childhood
Source: Sci Rep. 2017 May 12;7:1820. doi: 10.1038/s41598-017-02067-7 (PMC5431806; doi:10.1038/s41598-017-02067-7)
Supplement: Supplementary file 1 — Supplementary Information [file 41598_2017_2067_MOESM1_ESM.pdf]

**Supplementary information**

# Airway Microbial Diversity is Inversely Associated with Mite-Sensitized Rhinitis and Asthma in Early Childhood

Chih-Yung Chiu, Yi-Ling Chan, Yu-Shuen Tsai, Ssu-An Chen, Chia-Jung Wang, Kuan-Fu Chen & I-Fang Chung

Correspondence and requests for materials should be addressed to C.-Y.C. (email: pedchestic@gmail.com) or I.-F.C. (email: cifldmy@gmail.com)

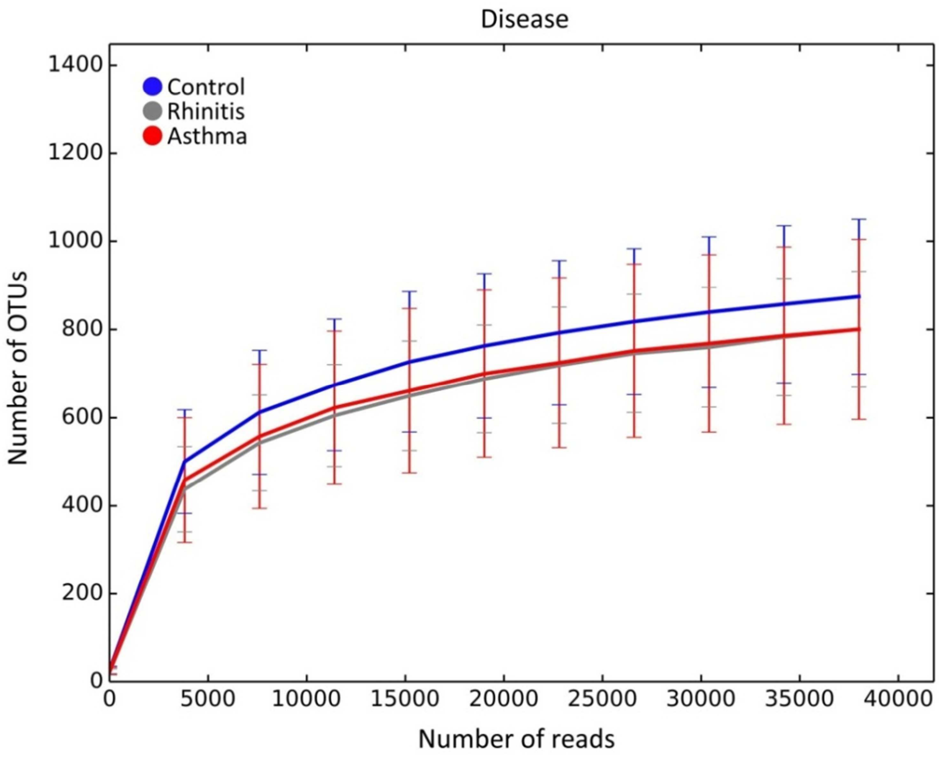


**Supplementary Figure S1. Rarefaction curves illustrating the degree of sample coverage in children with asthma, rhinitis and the healthy controls.** Rarefaction curves show the number of reads and their corresponding number of OTUs, and healthy children appear to have higher species richness than those with asthma and rhinitis.

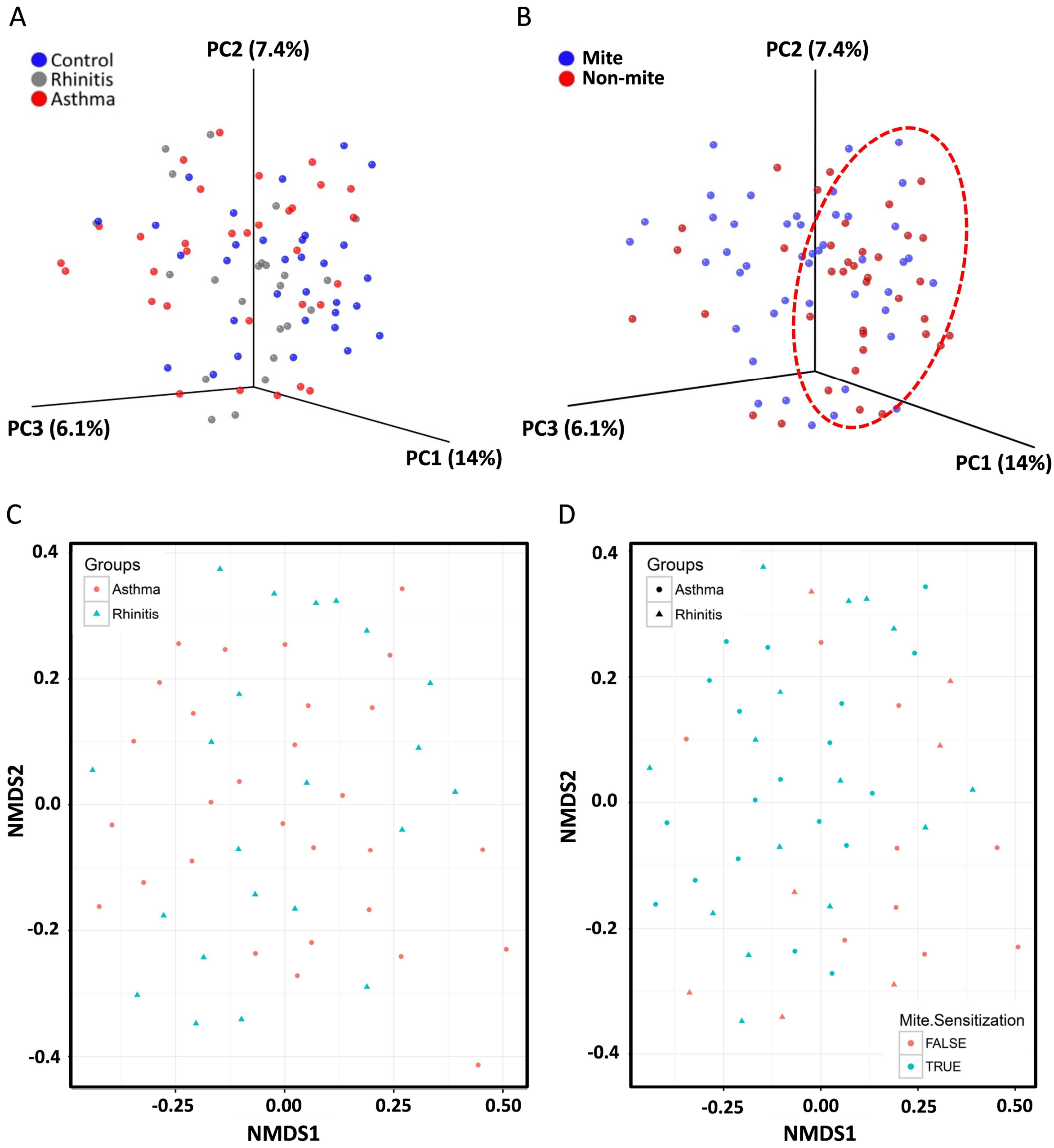


**Supplementary Figure S2. Beta diversity statistics using the Principal Coordinate Analysis (PCoA) and non-metric multidimensional scaling (NMDS).** Unweighted UniFrac PCoA plot comparing among rhinitis, asthma and healthy controls (A) and between with/without mite sensitization (B). RLB NMDS plot comparing between rhinitis and asthma (C) and between with/without mite sensitization (D). Each dot represents a sample. The results show no cluster patterns different between allergic airway diseases but mite sensitization (Adonis $P = 0.003$).
